# Medical student flourishing before and during the COVID-19 pandemic at one U.S. institution

**DOI:** 10.12688/mep.19094.1

**Published:** 2022-04-21

**Authors:** Margot Kelly-Hedrick, Kayla Iuliano, Sean Tackett, Margaret S. Chisolm

**Affiliations:** 1 Duke University School of Medicine, Durham, NC, USA; 2 Johns Hopkins Bloomberg School of Public Health, Baltimore, MD, USA; 3 Medicine, Johns Hopkins University School of Medicine, Baltimore, MD, USA; 4 Psychiatry and Behavioral Sciences, Johns Hopkins University School of Medicine, Baltimore, MD, USA

**Keywords:** flourishing, burnout, quality of life, empathy, undergraduate medical education, COVID-19, coronavirus-19

## Abstract

**Introduction: **Medical education research often focuses on measuring negative mental states like burnout, rather than focusing on positive states like well-being. Flourishing – a state that includes domains of happiness and mental health - is a way of thinking about well-being that may be relevant to education and research. The purpose of this prospective, observational study was to compare the relationship among flourishing, other well-being measures, and burnout in medical students via a survey administered at two time points.

**Methods: **We surveyed medical students at one U.S. institution about their flourishing, satisfaction with work-life balance, quality of life, empathic concern, and burnout (emotional exhaustion and depersonalization) before and after the onset of the COVID-19 pandemic. Flourishing was measured using two scores, the Flourish Index (FI) and Secure Flourish Index (SFI), with higher scores indicating greater flourishing. Pre- and post-scores for both measures were compared.

**Results: **107/585 (18%) medical students responded to the survey and 78/107 (73%) participated in the post survey. SFI scores were higher at the second time point (M=7.1, SD=1.2) than the first (M=6.7, SD=1.3, p=.026). FI, satisfaction with work-life balance, quality of life, empathic concern, and burnout were unchanged at the second time point.

**Discussion: **COVID-19 has disrupted medical students and their education in multiple ways – some of them positive – which may explain the increase in SFI score and the lack of change in FI and other measures, at the post-survey.

## Introduction

Nearly half of all medical students experience burnout ^1, 2^. One way of understanding burnout, a state when one’s sense of well-being goes awry, is by understanding well-being itself ^3^. Flourishing has been defined by VanderWeele as “a state in which all aspects of a person’s life are good,” emphasizing that well-being is marked by more than the absence of negative thoughts and feelings ^4^. Flourishing encompasses a broad set of positive components, including the domains of happiness and life satisfaction, physical and mental health, meaning and purpose, character and virtue, and close social relationships. In addition to these positive components, one needs a certain degree of financial security to achieve them ^4, 5^. VanderWeele and his team have attempted to capture and quantify the construct of flourishing—including the instrumental financial security—in a measure that has been studied in multiple, diverse samples from the general population ^6, 7^.

We have previously administered the flourishing measure to graduate medical trainees to explore the cross-sectional relationship among flourishing, other well-being measures, and burnout ^8^. That study found that higher levels of flourishing - as reported on the flourishing measure - correlated with higher quality of life and satisfaction with work-life balance, as well as lower levels of depersonalization and emotional exhaustion—well-established domains of burnout ^9^. Those results suggest the flourishing measure may be useful as a means of exploring various aspects of trainees’ mental health.

To our knowledge, this relationship has not yet been evaluated in undergraduate medical learners. Nor has this relationship been studied longitudinally in any group of medical learners. In addition, no reports of flourishing in medical learners before and during the COVID-19 pandemic have been published, although evaluation of other measures of medical student well-being during COVID-19 have been published, with mixed results ^10– 12^. We initially aimed to compare the cross-sectional and longitudinal relationship among flourishing, other well-being measures, and burnout in medical students via a survey administered at two time points. The unanticipated onset of the COVID-19 pandemic in the United States occurred between the survey’s two time points. The initial survey data were collected before the pandemic’s onset; and the follow-up survey data, when the pandemic had disrupted medical education and daily life in the U.S. Thus, the intervening onset of the pandemic between the two surveys allowed us unexpectedly to evaluate the impact of the pandemic on medical student flourishing.

## Methods

### Sample

We invited all current Johns Hopkins University medical students via email to participate in the study, which consisted of a similar survey administered at two time points. We sent reminder emails following the initial email at one, four, five, and eight weeks. Data collection for the initial survey took place from December 2019 to mid-February 2020. We emailed the follow-up survey in May 2020 to all medical students who had completed the initial survey and sent reminders after one, two, and four weeks. The survey was hosted on the online platform, Qualtrics, which is similar to free survey programs such as SurveyMonkey. Upon completion of both surveys, 78 participants were eligible to enter a raffle for a $25 gift card with a 1/25 chance of winning.

### Measures

Both surveys queried participants about flourishing, satisfaction with work-life balance, quality of life, empathic concern, and burnout (emotional exhaustion and depersonalization). These measures of well-being and burnout have been used in previous studies of medical learners ^8, 13– 17^. The initial survey also asked general demographic questions.

*Flourishing.* We measured flourishing using the flourishing measure, a short 12-item measure developed by VanderWeele that assesses six domains of flourishing: (1) happiness and life satisfaction, (2) physical and mental health, (3) meaning and purpose, (4) character and virtue, (5) close social relationships, and (6) financial and material stability. The sixth domain is included to assess resources to assist with achieving flourishing in the first five domains. Each item is scored on a 0–10 scale. The first ten items, pertaining to domains 1–5, are averaged to create a Flourish Index (FI) score. The financial and material stability items (domain 6) are included in the average to create a Secure Flourish Index (SFI) score ^5^.

*Satisfaction with work-life balance.* We assessed satisfaction with work-life balance via participants’ responses to the single item: ‘‘How satisfied are you with the balance between your personal and professional life?’’ on a Likert scale from 1 (very dissatisfied) to 5 (very satisfied). We defined low satisfaction with work-life balance as a response of 2 or lower, consistent with previous studies ^8, 13^.

*Quality of life.* We determined students’ quality of life with a single question: “Which of the following best describes your overall quality of life?” on a Likert scale, ranging from 1 (“as bad as it can be”) to 5 (“as good as it can be.”). We defined low quality of life as a response of 2 or lower, consistent with previous studies ^8, 13^.

*Empathic concern.* We measured empathic concern using the subscale from the Interpersonal Reactivity Index (IRI) ^17^. Each participant was asked to rate seven statements on a Likert scale (e.g., “I am often quite touched by things that I see happen.”) from 0 (does not describe me well) to 4 (describes me well) and responses are summed to create a total score (range 0 to 28) ^18^.

*Burnout (emotional exhaustion and depersonalization).* We assessed burnout using two single items modified from the Maslach Burnout Inventory (MBI), consistent with previous medical education studies ^8, 13, 14, 18^. These items asked participants to report the frequency of experiencing emotional exhaustion (‘‘How often do you feel burned out from your work and studying?’’) and depersonalization (‘‘How often do you feel you’ve become more callous toward people since you started medical school?’’) on a 7-point Likert scale, with options spanning “never” to “daily” ^18^. We considered participants who reported experiencing symptoms at least once a week to have high emotional exhaustion or high depersonalization, consistent with previous studies ^8, 13, 14^.

### Analysis

We tabulated descriptive statistics to summarize our respondent population. Cronbach’s alphas were calculated to determine internal consistency for the FI and SFI. We used paired t-tests to compare differences in means for flourishing measure individual items, FI and SFI scores, and for empathic concern totals. We used McNemar’s test for pre-post paired comparisons between dichotomized variables.

Statistical significance was set using a type I error threshold of alpha < .05. We present unadjusted p values. For comparisons for the 12 items on the flourishing measure, Bonferroni correction requires a p < .004 to achieve a type 1 error rate of < .05. For all other comparisons, statistical significance was set at p<.05. We completed all analysis in Stata 13 (StataCorp. 2013. Stata Statistical Software: Release 13. College Station, TX, USA. StataCorp LP).

### Ethical considerations

The Johns Hopkins Medicine Institutional Review Board reviewed the research study’s protocol (IRB00232184) and deemed it exempt under the DHHS regulations; and a waiver of consent was granted (decision date: Dec 6, 2019).

## Results

Of the 585 medical students invited to participate in the study, 107 completed the initial survey (response rate=18%). Of these 107,78 completed the follow-up survey (response rate=73%). Table 1 presents the demographic characteristics of this sample. Female, white, and first- and second-year medical students were overrepresented in our sample compared to the overall demographic data for students from Johns Hopkins School of Medicine.

**Table 1.** Sample demographics of participants who completed initial survey (n=105).

Characteristic	n (%)
Medical school year classification	
First	31 (30)
Second	26 (25)
Third year	13 (12)
Fourth year	18 (17)
Masters, PhD, or certificate training	17 (16)
Gender	
Female	71 (68)
Male	31 (29)
Non-binary/other	3 (3)
Race/ethnicity	
Caucasian	58 (55)
Asian	27 (26)
African American	7 (7)
Mixed ethnicity	8 (7)
East Indian	5 (5)
Pet Ownership	
Pet owner	35 (33)
Not a pet owner	70 (67)

Note. Percentages are based on participants who completed demographics information (n=105)

Table 2 displays the initial and follow-up survey values for the flourishing measure individual items and FI and SFI scores. Both the FI (10 items, alpha=.87 for initial, alpha=.88 for follow-up) and SFI (12 items, alpha=.83 for initial, alpha=.85 for follow-up) had good internal consistency. One item (concern about meeting monthly living expenses) increased significantly from initial (M=6.5, SD=3.3) to follow-up survey (M=7.7, SD=2.6), indicating *less* concern at the second time point (p=.0039).

**Table 2.** Mean and standard deviation of flourishing measure items, FI, and SFI scores at initial and follow up time points (n=78).

Item	Initial Mean (SD)	Follow-up Mean (SD)
Overall, how satisfied are you with life as a whole these days?	6.8 (1.5)	6.6 (1.7)
In general, how happy or unhappy do you usually feel?	6.4 (1.5)	6.2 (1.7)
In general, how would you rate your physical health?	6.3 (1.7)	6.7 (1.9)
How would you rate your overall mental health?	6.1 (1.7)	6.2 (1.9)
Overall, to what extent do you feel the things you do in your life are worthwhile?	7.2 (2.2)	7.3 (1.8)
I understand my purpose in life.	6.7 (2.5)	6.9 (2.2)
I always act to promote good in all circumstances, even in difficult and challenging situations.	7.7 (1.7)	7.7 (1.3)
I am always able to give up some happiness now for greater happiness later.	7.2 (1.9)	7.7 (1.7)
I am content with my friendships and relationships.	6.6 (2.2)	7.2 (1.7)
My relationships are as satisfying as I would want them to be.	6.4 (2.3)	6.8 (2.0)
**Flourish Index total score**	**6.7 (1.3)**	**6.9 (1.3)**
How often do you worry about being able to meet normal monthly living expenses?	6.5 (3.3)	7.7 (2.6) ^*a^
How often do you worry about safety, food, or housing?	7.1 (2.9)	7.8 (2.6)
**Secure Flourish Index total score**	**6.7 (1.3)**	7.1 (1.2) ^*b^

Note. For individual item analysis, significance value adjusted to p=.004 for multiple comparisons using Bonferroni correction. For the FI and SFI, significance was set at p<.05 and is denoted by an *. ^a^p=.0039. ^b^p=.026. For complete measure with scale anchors and scoring details see https://hfh.fas.harvard.edu/measuring-flourishing.

Table 3 presents initial and follow-up****** measures of FI, SFI, satisfaction with work-life balance, quality of life, empathic concern, emotional exhaustion, and depersonalization. Of these measures, only the SFI changed significantly between the initial and follow-up surveys; the mean SFI was higher at the second time point (M=7.1, SD=1.2) than the first (M=6.7, SD=1.3, p=.026).

**Table 3.** Differences in FI, SFI, and well-being measures at follow up survey (n=78).

Variable	All Initial (n=105)	Initial matched to follow-up (n=78) M (SD)	Follow-up (n=78) M (SD)	p value for difference ^1^
FI, M (SD)	6.9 (1.4)	6.7 (1.3)	6.9 (1.3)	.199
SFI, M (SD)	6.9 (1.3)	6.7 (1.3)	7.1 (1.2)	.026*
Low satisfaction with work-life balance, n (%)	28 (26)	23 (29)	23 (29)	1.00
Low quality of life, n (%)	9 (8)	7 (9)	5 (6)	.53
Empathic concern (IRI), M (SD)	22.1 (4.2)	22.2 (4.0)	21.8 (4.6)	.130
High depersonalization, n (%)	19 (18)	15 (19)	12 (15)	.37
High emotional exhaustion, n (%)	42 (39)	31 (40)	31 (40)	1.00

^1^Paired t-test (initial/follow-up) for continuous outcomes (FI, SFI, IRI) and McNemar’s Chi-squared statistic for categorical variables (work-life balance, quality of life, depersonalization, emotional exhaustion). Significance set at p<.05.

Table 4 presents differences in FI and SFI between participants who endorsed low satisfaction with work-life balance, low quality of life, high emotional exhaustion, and high depersonalization—each compared to participants who reported the opposite in each respective category. All reported data are from the initial survey collection, prior to the onset of the pandemic. These data were unchanged between the initial and follow-up assessments, with the exception that emotional exhaustion was no longer significantly related to FI or SFI at the follow-up time point.

**Table 4.** Mean FI and SFI scores by well-being variables at initial time-point (n=105).

Variable	Mean FI (SD)	p-value	Mean SFI (SD)	p-value
**Satisfaction with work-life balance**				
Low satisfaction	5.8 (1.4)	<.001 ^***^	6.0 (1.3)	<.001 ^***^
Higher satisfaction	7.1 (1.1)		7.1 (1.1)	
**Quality of life (QOL)**				
Low QOL	4.5 (1.2)	<.001 ^***^	4.7 (1.1)	<.001 ^***^
Higher QOL	6.9 (1.1)		6.9 (1.1)	
**Emotional exhaustion**				
High emotional exhaustion (≥ weekly)	6.0 (1.5)	<.001 ^***^	6.2 (1.5)	.004 ^**^
Less emotional exhaustion (< weekly)	7.2 (0.9)		7.1 (1.1)	
**Depersonalization**				
High depersonalization (≥ weekly)	6.0 (1.3)	.021 ^*^	6.1 (1.1)	.023 ^*^
No depersonalization (< weekly)	6.9 (1.3)		6.1 (1.1)	

Note. Significance set at p<.05. *=p<.05, **=p<.01, ***=p<.001.

## Discussion

We collected data on medical student flourishing, satisfaction with work-life balance, quality of life, empathic concern, and burnout at one U.S. medical school across two points—one before and one after the onset of the COVID-19 pandemic. Overall, we found medical students reported no change in flourishing, and that measures of flourishing correlated with the other measures of well-being and burnout.

We found no significant changes in the FI, satisfaction with work-life balance, quality of life, empathic concern, or burnout at the second time point compared to the first. Some studies have found medical students report mental health deterioration during COVID-19 ^19– 22^, while others have found more nuanced results ^21, 23, 24^. One survey of medical students in Cyprus before and during the pandemic found no change in burnout ^21^. A qualitative study of Australian medical students identified areas that the pandemic negatively impacted life (e.g., stress, studies), but some students also pointed out some aspects of life, such as family, exercise, finances, and sleep were positively affected ^24^. Similarly, a study of first year osteopathic medical students found that students varied greatly in how their life and functioning was impacted by the transition to online classes due to COVID-19 ^23^. These studies took place in different countries, health systems, and time points—and likely impacted different students in different ways, which may not be adequately captured by a quantitative, close ended measure.

Data from the general population between January and June 2020 showed a decrease in flourishing overall, though not even domain was equally affected. The largest drop in score was for the financial and material stability domain ^25^. In contrast, our study found the SFI, which included the financial and material stability questions, and the item on ability to meet monthly expenses, were the only significant changes in a positive direction, suggesting some students worried *less* about finances during the onset of the pandemic than before. This could possibly be due to students leaving campus and returning to live with their families or fewer daily expenditures (e.g., transportation); however, we were not able to glean why this may be the case from our data.

We found that medical students with higher FI and SFI scores had higher satisfaction with work-life balance, higher quality of life, and lower levels of burnout. These associations were also found in our previous study piloting the measure with graduate medical trainees, further supporting the idea that the concept of flourishing is related to other relevant constructs ^7^. However, flourishing may be getting at a more holistic and useful concept to medical education than measuring negative states, which may leave us too narrowly focused ^3^.

Our study has several limitations. Our response rate for the initial survey was low and our sample was not representative of the medical school student body as a whole. The limited nature of our survey (i.e., close-ended) did not allow us to further explore how participants were being affected by the pandemic and resulting changes to their medical education. It is also possible that those students who did not respond to the follow up survey were more negatively impacted, making the mean flourishing scores higher than if these participants were included. We were unable to characterize how these measures may have changed as the pandemic further unfolded throughout 2020 and beyond.

We measured medical students’ flourishing, satisfaction with work-life balance, quality of life, empathic concern, and burnout before and during the COVID-19 pandemic at one institution. We found these variables remained largely unchanged after the pandemic onset. Future research could further explore how the flourishing measure may change in different settings and circumstances.

## Data availability

### Underlying data

As the ethical approval and participant consent process required, the data underlying the findings of the study cannot be made publicly available due to their containing information that could compromise privacy of research participants. Due to the small sample size, with disclosure in the paper of which medical school the students are from, responses may be identifiable based on their demographic and participant characteristics responses. However, the data are available upon reasonable request to Dr. Margaret S. Chisolm [ mchisol1@Jhmi.edu] under the conditions that the writer describes their purpose for gaining access to the data and intentions for using the data.
