# Supplementary material for: Medical student flourishing before and during the COVID-19 pandemic at one U.S. institution
Source: MedEdPublish (2016). 2023 Mar 3;12:28. Originally published 2022 Apr 21. [Version 2] doi: 10.12688/mep.19094.2 (PMC10039319; doi:10.12688/mep.19094.2)
Supplement: Peer review history

# Peer review: Reviewer response for version 2

**DOI:** 10.21956/mep.20987.r32990

**Published:** 2023-03-24

**Authors:** Michael Jibson

**Affiliations:** 1 University of Michigan, Ann Arbor, MI, USA

I appreciate the opportunity to review this revised manuscript. The authors have taken the additional steps of including and discussing the overall data from the medical school and although that confirmed that their sample was skewed demographically, the study methodology, if not the conclusions, is strengthened by this information. 

 As I mentioned previously, the study has merit primarily in its introduction of measures of flourishing as a valid indicator of student well-being. The manuscript achieves that and looks fine in the current form.

Have any limitations of the research been acknowledged?

Yes

Is the work clearly and accurately presented and does it cite the current literature?

Yes

If applicable, is the statistical analysis and its interpretation appropriate?

Yes

Are all the source data underlying the results available to ensure full reproducibility?

Yes

Is the study design appropriate and is the work technically sound?

Yes

Are the conclusions drawn adequately supported by the results?

Partly

Are sufficient details of methods and analysis provided to allow replication by others?

Yes

Reviewer Expertise:

Medical education (competency assessment)

I confirm that I have read this submission and believe that I have an appropriate level of expertise to confirm that it is of an acceptable scientific standard.

# Peer review: Reviewer response for version 1

**DOI:** 10.21956/mep.20452.r32393

**Published:** 2022-08-16

**Authors:** Michael Jibson

**Affiliations:** 1 University of Michigan, Ann Arbor, MI, USA

This is an interesting and timely study with the primary purpose of validating a positive measure of medical student wellness, "flourishing," as an alternative to the negative measures (e.g., "burnout") that are so prevalent in LCME- and ACGME-accredited programs. Coincidentally, the follow-up study overlapped the onset of the COVID pandemic, allowing an assessment of the pandemic's impact on students' well-being. Both of these are worthwhile topics, but I especially commend the first, as the current incessant focus on negative measures may be worsening trainees' well-being by its constant demand that negative issues be examined.

 The basic design of the study, including the statistical analysis, was straightforward and appropriate.

 The greatest weakness of the study was its small and skewed sample. The authors candidly acknowledged this problem and the likelihood that the self-selection of participants skewed the results, as well, noting that struggling students may be less willing or feel less able to sacrifice the extra time and effort required to do the survey. That rather glib acknowledgement may not be sufficient in all readers' minds to address the issue. One simple step to mitigate this somewhat would be to add a column to Table 1 with the demographics of the entire medical school student body. More convincing would be data from the dean's office regarding measures of burnout of the student body to compare with those measures in the study sample.

 Even in the absence of those additional data, this non-random sample of students still has merit in its correlation with negative measures, allowing at least a preliminary conclusion that "flourishing" is a valid quality that can be reliably measured and meaningfully interpreted, making it a useful tool for educators interested in trainee well-being.

 Finally, I commend the authors on the quality of the writing in this report, which is exemplary in its clarity, flow, organization, and reasoning, making it a pleasure to read. Having said that, there is an apparent typo in paragraph 3 of the discussion, which substituted "even" for "every," (at least the sentence makes more sense that way).

Have any limitations of the research been acknowledged?

Yes

Is the work clearly and accurately presented and does it cite the current literature?

Yes

If applicable, is the statistical analysis and its interpretation appropriate?

Yes

Are all the source data underlying the results available to ensure full reproducibility?

Yes

Is the study design appropriate and is the work technically sound?

Yes

Are the conclusions drawn adequately supported by the results?

Partly

Are sufficient details of methods and analysis provided to allow replication by others?

Yes

Reviewer Expertise:

Medical education (competency assessment)

I confirm that I have read this submission and believe that I have an appropriate level of expertise to confirm that it is of an acceptable scientific standard, however I have significant reservations, as outlined above.

# Peer review: Reviewer response for version 1

**DOI:** 10.21956/mep.20452.r32245

**Published:** 2022-07-18

**Authors:** Amy Farkas

**Affiliations:** 1 Division of General Internal Medicine, Department of Medicine, Medical College of Wisconsin, Milwaukee, WI, USA

The authors present an interesting article on flourishing in medical students that demonstrates that the Flourishing Index does correlate with other commonly used metrics for wellbeing such as quality of life and burnout measures like their cited work in the GME population. This is a useful finding as it helps to establish the use of the Flourishing Index as a more wholistic measure of medical trainees. While the authors were able to assess for the impact of the COVID-19 pandemic based on the timing of their study, I find the correlation of the index with other measures a more significant take home point of the work. The authors did not find any change in the flourishing index after the onset of the pandemic and highlight that the impact of COVID-19 on medical student well-being is mixed in the literature. Additionally, because the study was not initial designed to measure the impact of COVID-19 on medical student well-being the authors did not have the opportunity to assess other life factors that may have changed secondary to the pandemic. Finally, the second time point was in May of 2020, which may not fully capture the impact of the pandemic on medical students’ well-being which likely changed overtime.

 The methodology of the study is appropriate as is the statistical analysis. The results are presented clearly, and the tables are easy to interpret. While the convenience sample is a limitation to the work the authors acknowledge this limitation appropriately. The discussion presents the findings as it relates to the known literature and the conclusions are supported by the data.

 Overall I do not have any significant revisions for this work.

Have any limitations of the research been acknowledged?

Yes

Is the work clearly and accurately presented and does it cite the current literature?

Yes

If applicable, is the statistical analysis and its interpretation appropriate?

Yes

Are all the source data underlying the results available to ensure full reproducibility?

Yes

Is the study design appropriate and is the work technically sound?

Yes

Are the conclusions drawn adequately supported by the results?

Yes

Are sufficient details of methods and analysis provided to allow replication by others?

Yes

Reviewer Expertise:

Medical education, mentorship, women's health

I confirm that I have read this submission and believe that I have an appropriate level of expertise to confirm that it is of an acceptable scientific standard.
